# Systematic review of birth cohort studies in Africa

**Published:** 2011-06

**Authors:** Alasdair Campbell, Igor Rudan

**Affiliations:** 1 Centre for Population Health Sciences and Global Health Academy, University of Edinburgh, Scotland, UK; 2 Croatian Centre for Global Health, University of Split School of Medicine, Split, Croatia

## Abstract

**Aim:**

In sub-Saharan Africa, unacceptably high rates of mortality amongst women and children continue to persist. The emergence of research employing new genomic technologies is advancing knowledge on cause of disease. This review aims to identify birth cohort studies conducted in sub-Saharan Africa and to consider their suitability as a platform to support genetic epidemiological studies.

**Methods:**

A systematic literature review was conducted to identify birth cohort studies in sub-Saharan Africa across the following databases: MEDLINE, EMBASE, AFRO and OpenSIGLE. A total of 8110 papers were retrieved. Application of inclusion/exclusion criteria retained only 189 papers, of which 71 met minimum quality criteria and were retained for full text analysis.

**Results:**

The search revealed 28 birth cohorts: 14 of which collected biological data, 10 collected blood samples and only one study collected DNA for storage. These studies face many methodological challenges: notably, high rates of attrition and lack of funding for several rounds of study follow up. Population-based ‘biobanks’ have emerged as a major approach to harness genomic technologies in health research and yet the sub-Saharan African region still awaits large scale birth cohort biobanks collecting DNA and associated health and lifestyle data.

**Conclusion:**

Investment in this field, together with related endeavours to foster and develop research capacity for these studies, may lead to an improved understanding of the determinants of intrauterine growth and development, birth outcomes such as prematurity and low birth weight, the links between maternal and infant health, survival of infectious diseases in the first years of life, and response to vaccines and antibiotic treatment.

The last two decades have seen a dramatic rise in research output in longitudinal birth cohort studies (**Figure 1**) (1). Several large quality birth cohort studies have been conducted, and are still ongoing, in high income countries (2-11). These studies provide useful insight into the developmental, social and environmental exposures that interact in determining disease risk (4-6) and they have led to many notable discoveries (7-9).

**Figure 1:**
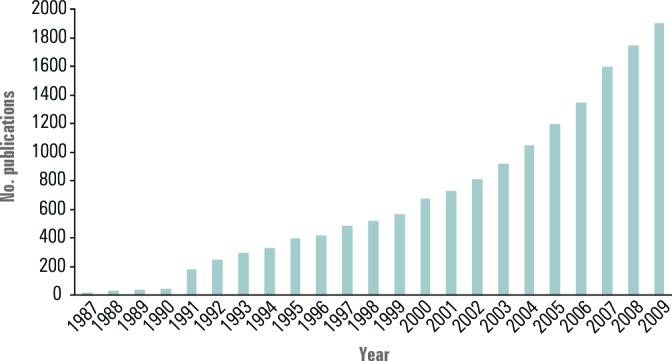
Number of publications resulting from a search of the ISI *Web of Science* database for any article containing the term ‘birth cohort study’ in the past 20 years; no language or other restrictions.

In recent years, large ‘biobank’ studies have emerged as the most successful way of harnessing new genomic technologies, with the aim of providing resources for the future investigation of the separate and combined effects of genetic, environmental and lifestyle factors underlying multifaceted human diseases (10). The value of combining epidemiological and genomic data by using large scale cohort study design is growing in recognition and studies recruiting more than 500 000 participants are already established or under way. These studies, such as the UK Biobank (11), have focused mainly on studying the major common diseases of public health importance, amongst adults in the developed world.

Many diseases of the poor, which represent the greatest health burden in terms of global mortality, have largely been neglected from this field of research. In 2008 there were an estimated 8.8 million child deaths 5.97 million (68%) were caused by infectious diseases and nearly half (4.20 million) of the deaths occurred in Africa (12). Global child mortality has fallen since 1990, yet the targets outlined in the Millennium Development Goals to reduce this by two thirds before 2015, are not being met by many countries (13). In sub-Saharan African (SSA) countries, maternal complications of pregnancy and communicable diseases of women and children are still major public health concerns, with an unacceptably high burden of mortality (2,12,13).

A study by Moran et al (14), at the Institute for International Health in Sydney, observed a marked discrepancy between funding for research and development relative to disease burden. This mismatch (with very low research investment relative to disease burden) was most notable for bacterial pneumonia and diarrhoea which account for 18% and 15% of global child deaths, respectively, most of these deaths being concentrated in several large developing countries in SSA and South East Asia (12). In addition to this, the transferability of research findings on determinants of disease from high-income countries to SSA may be limited. For example, between African and European studies different distributions of genetic polymorphism have been found which determine genetic susceptibility to both communicable disease (eg, malaria, HIV, tuberculosis) and non-communicable disease (eg, breast and prostate cancer) (15). Birth cohort studies conducted in high income countries fail to represent the conditions in poverty stricken areas of the world and there is a clear need for broader geographical representation in this area of research.

The aims of this review were 3-fold:

1. to provide a systematic review of birth cohort studies from SSA and discuss some important characteristics of these studies (population size, length of study, and follow up frequency);

2. to examine the methodology of and the data collected from birth cohort studies in SSA;

3. to offer recommendations on the feasibility and sustainability of support for this area of research based on the findings of this review and in the context of the existing literature.

## METHODS

### Search strategy

**Figure 2:**
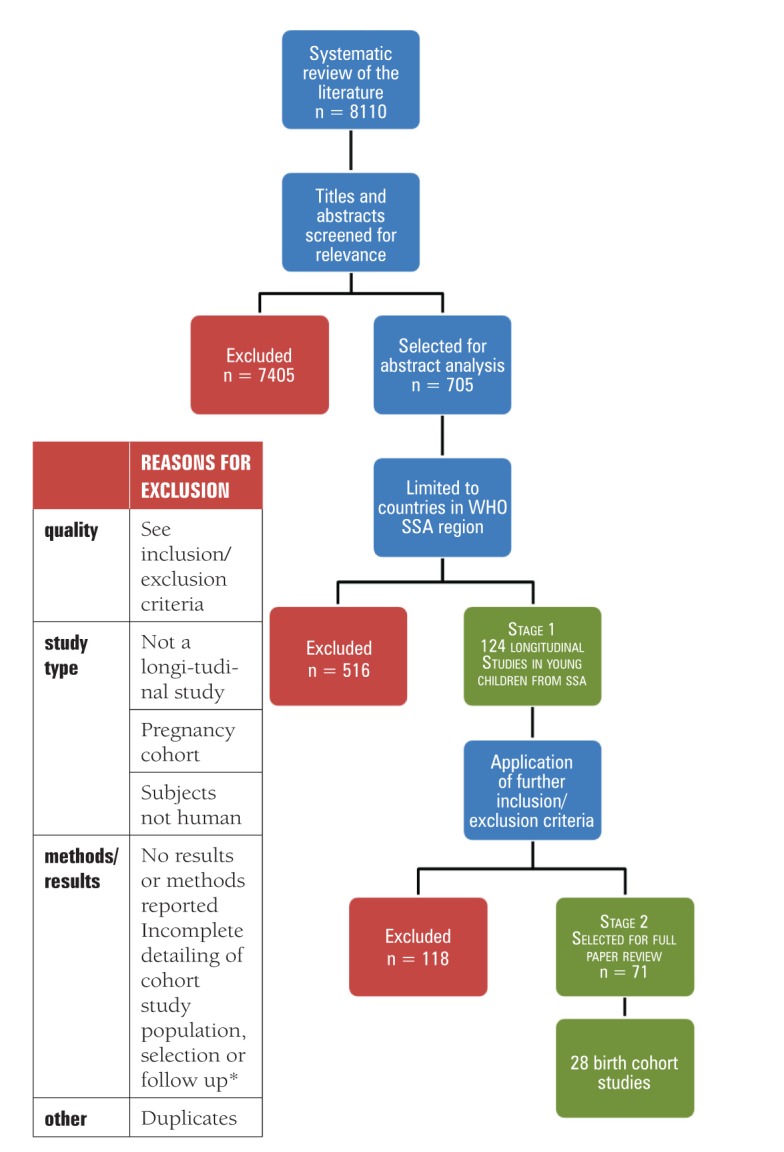
Summary of the literature search.

After initial scoping exercises and input from a librarian to provide MeSH headings and keywords pertinent to this study, a systematic search was conducted across the following databases (**Figure 2**):

1) via OVID: Medline (1950 onwards) on 30 July 2010 and Embase (1980 onwards) on 19 August 2010, using the strategy outlined in **Table 1**;

**Table 1:** Search strategy for Medline and Embase*

**Birth cohort** 1. (birth* adj3 cohort*).ti,ab. 2. (pregnan* adj3 cohort*).ti,ab. 3. famil* cohort*.ti,ab. 4. (birth* adj3 longitudinal.ti,ab.	**OR**	**Longitudinal studies** 5. Longitudinal Studies/ 6. cohort* survey*.ti,ab. 7. panel stud*.ti,ab. 8. panel survey*.ti,ab.
**AND**		
**Developing countries** 1. exp Developing Countries/ 2. africa/ or africa, northern/ or algeria/ or egypt/ or libya/ or morocco/ or tunisia/ or “africa south of the sahara”/ or africa, central/ or cameroon/ or central african republic/ or chad/ or congo/ or “democratic republic of the congo”/ or equatorial guinea/ or gabon/ or africa, eastern/ or burundi/ or djibouti/ or eritrea/ or ethiopia/ or kenya/ or rwanda/ or somalia/ or sudan/ or tanzania/ or uganda/ or africa, southern/ or angola/ or botswana/ or lesotho/ or malawi/ or mozambique/ or namibia/ or south africa/ or swaziland/ or zambia/ or zimbabwe/ or africa, western/ or benin/ or burkina faso/ or cape verde/ or cote d'ivoire/ or gambia/ or ghana/ or guinea/ or guinea-bissau/ or liberia/ or mali/ or mauritania/ or niger/ or nigeria/ or senegal/ or sierra leone/ or togo/ or “antigua and barbuda”/ or cuba/ or dominica/ or dominican republic/ or grenada/ or guadeloupe/ or haiti/ or jamaica/ or “saint kitts and nevis”/ or saint lucia/ or “saint vincent and the grenadines”/ or central america/ or belize/ or costa rica/ or el salvador/ or guatemala/ or honduras/ or nicaragua/ or panama/ or panama canal zone/ or mexico/ or argentina/ or bolivia/ or brazil/ or chile/ or colombia/ or ecuador/ or guyana/ or paraguay/ or peru/ or suriname/ or uruguay/ or venezuela/ or asia, central/ or kazakhstan/ or kyrgyzstan/ or tajikistan/ or turkmenistan/ or uzbekistan/ or cambodia/ or east timor/ or indonesia/ or laos/ or malaysia/ or myanmar/ or philippines/ or thailand/ or vietnam/ or asia, western/ or bangladesh/ or bhutan/ or india/ or sikkim/ or afghanistan/ or iran/ or iraq/ or jordan/ or lebanon/ or syria/ or turkey/ or yemen/ or nepal/ or pakistan/ or sri lanka/ or exp china/ or korea/ or “democratic people's republic of korea”/ or “republic of korea”/ or mongolia/ or albania/ or lithuania/ or bosnia-herzegovina/ or bulgaria/ or byelarus/ or “macedonia (republic)”/ or moldova/ or montenegro/ or romania/ or russia/ or serbia/ or ukraine/ or yugoslavia/ or exp transcaucasia/ or armenia/ or azerbaijan/ or “georgia (republic)”/ or comoros/ or madagascar/ or mauritius/ or seychelles/ or fiji/ or papua new guinea/ or vanuatu/ or palau/ or samoa/ or tonga/ 10. low income countr*.tw. 11. middle income countr*.tw. 12. (low adj2 middle income countr*).tw.		

*Within each box all terms were combined with Boolean operator OR. Developing countries were those defined by the World Bank list of economies (July 2010) as low- or middle-income.

2) via the Global Health Library Regional Index: AFRO on 22 August 2010, using ‘cohort’ as a keyword;

3) search of grey literature: via OpenSIGLE on 22 August 2010, using ‘birth’ and ‘cohort’ as keywords.

An informal search of Google Scholar produced no additional results.

Reference lists of finally selected papers were hand searched for further studies.

The aim of this search was to identify all birth cohort studies and not necessarily all publications relating to each study.

### Inclusion/exclusion criteria

We defined a birth cohort study as a study collecting data from a group of people born at a similar time, by active (medical examinations etc) and/or passive (hospital records, etc.) surveillance, with follow up over a variable period of time (months to decades) (**Table 2**). Initial inclusion criteria were sensitive but not specific, so that we could retrieve longitudinal studies which, whilst not necessarily meeting strict definitions of birth cohort studies, are useful in providing an overview of the characteristics of studies collecting longitudinal data in infancy/childhood in SSA. For more specific birth cohort analysis, strict criteria were applied to studies in a follow-up to the initial assessment. Studies that met the criteria were retained for full text analysis in order to focus further on quantitative and qualitative aspects of data collection.

**Table 2:** Inclusion and exclusion criteria for assessing the studies relevant to birth cohorts in sub-Saharan Africa

Inclusion criteria	Exclusion criteria
**Stage 1** • birth cohort/ longitudinal cohort or cross-sectional study of longitudinal population **or** qualitative review of birth cohort. • low and middle income countries • reporting primary results or insight to methodology or meta-analysis of studies	**Stage 1** • enrolment at >10 y of age • primarily a pregnancy study limited to birth weight follow up
**Stage 2** • country in WHO South Saharan African Region	**Stage 2 –Quality criteria** • minimum of 12 mo of follow up • minimum cohort size of 500 • less than 60 mo of age at induction

### Data extraction

Studies included in Stage 1 were extracted to an Excel file and analysed by abstract alone. This exercise aimed to provide background perspective of longitudinal studies in SSA rather than provide comprehensive data extraction. Studies included for full paper review (Stage 2) were assessed in full and the data obtained was intended to be comprehensive.

Both qualitative and quantitative data were assessed. Studies were assessed by categories that included, but were not limited to, biological samples (such as blood, DNA and urine); anthropometric data; cognitive, psychological and other developmental indicators; socioeconomic data; methodological challenges; and attrition.

## RESULTS

**Figure 3:**
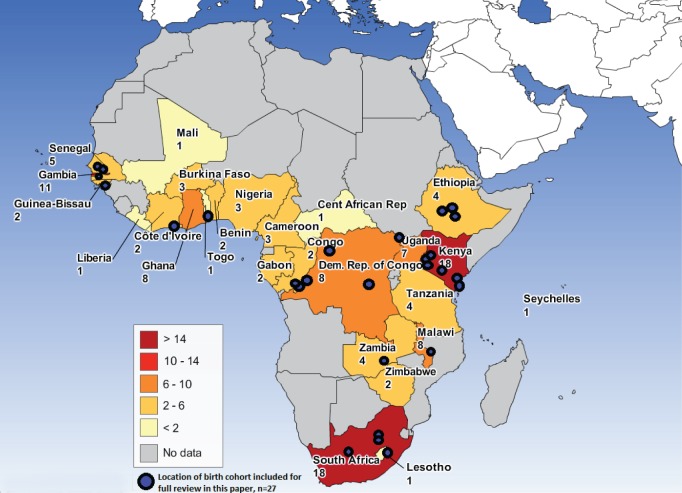
Number of birth cohort studies in Sub-Sahran Africa (SSA) by country. Coloured boxes refer number of studies in each country (out of 124 birth cohorts). Blue spots relate to the specific locations of the 28 cohorts which met full quality criteria. One paper was not presented due to multiple locations of study site.

Abstract analysis of 189 papers produced data from 124 longitudinal cohort studies (**Table 3** and **Figure 3**) meeting Stage 1 criteria. Abstracts were analysed for basic study characteristics (**Table 3**).

**Table 3:** Characteristics of 124 birth cohort studies in Sub-Saharan Africa (SSA)

Characteristics		No. of studies
Size of study*	0-500	49
	501-1000	22
	1001-1500	6
	1501-2000	6
	2001-2500	4
	>2500	11
	Not reported†	26
Maximum age at enrolment (months)	Antenatal-birth	53
	0-12	14
	13-24	5
	25-36	5
	37-48	3
	49-60	12
	>60	10
	Not reported†	22
Duration of follow up (months)	0-24	64
	25-48	13
	49-72	7
	73-96	1
	96-120	3
	>120	5
	Not reported†	31
Follow up frequency (months)	Daily	1
	Weekly	5
	Monthly	13
	2-6monthly	17
	>6monthly	15
	Not reported†	74
Setting	Community based	46
	Facility based	7
	Not reported†	71
Specific population	Born to HIV+ mother	19
	Malaria endemic area	9
	Malnourished/ underweight	6
	Intervention population	2
	Not specific	76
	Other	12

* Sometimes varied between publications.

† Details not presented in abstract.

### Study characteristics – Stage 1

*Study size:* The study sizes ranged from 30 to 11 342 participants. 79 of the studies recruited 1000 or less participants and only 27 studies recruited more than 1000 at induction. 59 recruited less than the quality criteria (500 participants) suggested in this study.

*Follow up:* 53 studies began data collection either in the antenatal period or at birth. The follow up period of participants ranged from 3 months to 20 years. The ‘Birth To Twenty’ study in South Africa is the longest running, has followed the initial cohort for 20 years and is still ongoing. The majority of the studies (n = 63), however, followed the cohort for 2 years or less.

*Frequency:* Frequency of follow up ranged from daily to two-yearly. Most abstracts did not report data on follow up frequency and full text analysis was needed.

*Methods:* 48 studies were identified that followed a specific population. The large majority of studies identified for setting were conducted in the community.

### Study characteristics – Stage 2

The study characteristics of the full paper reviews are found in **Table 4**. Analysis of 71 full text publications produced data for 28 separate birth cohort studies. All further results relate to these 28 studies.

**Table 4:** Characteristics of studies included for analysis

Reference	Country	Location	Year	Type of cohort study	Cohort size at enrolment	Maximum age at enrolment (months)	Follow up (months)	Follow up frequency	Attrition* (%)
Carme 1984; Guillo du Bodan 1984	Congo	Linzolo	1984†	retrospective	1003	birth	60	‡	‡
Carme 1992; Carme 1994	Congo	Linzolo	1981	retrospective	2424	birth	24	twice	‡
Adjorlolo-Johnson 1994	Cote d’Ivoire	Abidjan	1990	prospective	619	birth		3 monthly	4.4
Greenberg 1991	Democratic Republic of Congo	Kinshasa	1986	prospective	587	9	18	‡	4
Hauspie 1989	Democratic Republic of Congo	‡	1977	prospective	4030	48	48	‡	‡
Van Lerberghe 1986	Democratic Republic of Congo	Kasongo	1974	prospective	4273	60	12	‡	26
Ryder 1994a; Ryder 1994b	Democratic Republic of Congo	Kinshasa	1986	prospective	1091	birth	36	monthly	‡
Alemu 1996; Asefa 1996; Asefa 1998; Lesaffre 1999	Ethiopia	Jimma	1992	prospective	1563	birth	12	2 mon thly	14
Byass 2008	Ethiopia	Butajira	1987	prospective	1884	birth	216	monthly/ quarterly	‡
Lindtjorn 1992	Ethiopia	Dubluk/ Elka	1989	prospective	828	60	24	2 weekly	‡
Kristensen I 2000	Guinea-Bissau	‡	1990	prospective	8752	birth	20	‡	0†
Fegan 2007	Kenya	‡	2004	prospective	3500	59	24	yearly	‡
Malhotra 2009	Kenya	‡	2002	prospective	586	birth	36	6 monthly	34
McElroy 1999; McElroy 2000; McElroy 2001	Kenya	Asembo bay	1992	prospective	942§	birth	48	2 weekly	28
Nokes 2004; Ochola 2009	Kenya	Kilifi	2002	prospective	635	birth	12	monthly	10
Oomen 1979	Kenya	Machakos	1974	prospective	568	60	22	monthly	32
ter Kuile 2003; van Eijk 2002	Kenya	Kisumu	1996	prospective	661	0	12	monthly	46
Cohen 1976	Lesotho	‡	1969	cohort/ cross-sectional	1317	60	60	‡	‡
Maleta 2003	Malawi	Lungwena	1995	prospective	767	0	36	monthly	72.9
Chilongozi 2008	Malawi, Tanzania, Zambia	‡	2001	prospective	2383	0	12	2 monthly	44
Elguero 2005	Senegal	‡	1989	analysis of two birth cohorts	11342	0	24	‡	‡
Simondon 1998	Senegal	Niakhar	1983	mixed retrospective/ prospective	1650	60	120	twice	46.8
Kristensen 2006	South Africa	Soweto	2000	prospective	571	0	12	2 weekly	21
Reid 1990	South Africa	Prieska	1932	retrospective	1227	0 /birth record	48	twice	32.5
Adair 2009; Ellison 2000; Griffiths 2008; Heerden 2010; Jones 2008; MacKeown 2000; MacKeown 2003 MacKeown 2007; Martorell 2010; Naicker 2010; Norris 2009; Pedro 2008; Richter 1995; Richter 2006; Richter 2007; Sabet 2009; Sheppard 2009; Steyn 2000; Steyn 2006; Thandrayen 2009; Vidulich 2006; Whati 2005; van Yach 1991	South Africa	Johannesburg (Soweto)	1990	prospective	3273	antenatal period (mother) and birth	ongoing	yearly	28§
Rayco-Solon 2004	The Gambia	West Kiang	1950	retrospective	3981	0	death/ Oct 97	yearly	‡
Vella 1993; Vella 1994	Uganda	Arua	1987	prospective	1072	60	24	‡	‡
McDowell 1982	Zambia	‡	1970	prospective	1342	0	60	6 monthly	‡

PC  =  prospective cohort, RC  =  retrospective cohort

*Attrition at last recorded study of initial cohort. Attrition varied by study outcome.

†Publication year.

‡Data not reported.

§ Reporting of study size varies (1570, 942 and 833); 942 reported in first paper, so this value taken.

*Study size:* Of the 28 studies retained for full paper analysis, the median number of participants at induction is 1272, ranging from 571 to 11 342.

*Follow up:* Median follow up was 24 months, ranging from 12 to 216 months. Median frequency of follow up was bimonthly, ranging from bi-weekly to five annually.

*Age at induction:* The mean age of study participants at induction was 17 months.

### Biological measurements

*Blood:* Ten of the studies collected blood from study participants (**Table 5**). A number of these ten studies also took other blood samples, including: maternal blood (n = 8), cord blood (n = 5), placental blood (n = 3) and blood smears (n = 5). Analysis of samples varied. The most common tests performed on samples were ELISA and Western blot for HIV status and Giemsa stain for malaria.

**Table 5:** An overview of the studies that collected biological samples

	Blood sample						Sample analysis	Frequency of sampling	
Reference	Maternal	Cord blood	Placental	Off-spring blood	Plasma/serum	Blood smear		Fixed	Ongoing
Adjorlolo-Johnson 1994	Y	N	N	Y	serum	N	ELISA, Western blot, synthetic peptide tests, flow cytometry, cell counts.	–	–
Greenberg 1991	Y	Y	N	Y	–	Y	Screen for HIV-1 antibodies by ELISA, confirmed by Western blot. Cord-blood from 64 randomly selected seropositive infants.	12 and 18 months of age	–
Ryder 1994a; Ryder 1994b	Y	N	N	Y	serum	N	ELISA, Western blot.	–	–
Malhotra 2009	Y	Y	Y	Y	Y	Y	Immunoglobulims, antibodies, filter for Wuchereria bancrofti microfilariae, Hb levels (HemoCue). Antigens, mitogens and recombinant cytokines.	Birth	Six monthly
McElroy 1999; McElroy 2000; McElroy 2001	Y	Y	Y	Y	plasma	Y	Hb levels: “hemocue”, Microscopy: Giemsa	Birth	Monthly (finger prick) Two weekly (blood film)
Nokes 2004; Ochola 2009	N	Y	N	Y	–	N	ELISA	Birth	Three monthly
ter Kuile 2003; van Eijk 2002	Y	N	Y	Y	–	Y	Finger prick for HIV test, malaria smear, Haemocue for hb, serostrip HIV-1/2 and Capillus HIV1-HIV2 tests	Pre-inclusion (maternal blood), pregnancy, 1 month post partum	–
Chilongozi 2008	Y	N	N	Y	–	N	Syphilis screen, ELISA or Western blot test, complete blood count, CD4 cell count, plasma viral load.	–	–
Adair 2009; Ellison 2000; Griffiths 2008; Heerden 2010; Jones 2008; MacKeown 2000; MacKeown 2003; MacKeown 2007; Martorell 2010; Naicker 2010; Norris 2009; Pedro 2008; Richter 1995; Richter 2006; Richter 2007; Sabet 2009; Sheppard 2009; Steyn 2000; Steyn 2006; Thandrayen 2009; Vidulich 2006; Whati 2005; van Yach 1991	Y	Y	N	Y	–	–	Non fasting sample- lipids. Lead, glucose, insulin, cotinine, HIV status, vit D and bone turnover markers.	Birth, 5 years, 13 years and 14 years.	
Rayco-Solon 2004	N	N	N	Y	–	Y			
	8	5	3	10		5			

Y – yes, N – no

*DNA:* most of the longitudinal studies in SSA did not take DNA samples. Five studies collected DNA: 3 were to assess HIV status, 1 to detect malaria and only 1 stored DNA. The ‘Birth to Twenty’ study in South Africa began DNA collection in 2005 and currently has samples for 2200 participants.

### Other biological samples

Very few other biological samples were taken by studies. Thirteen of the 28 studies collected no biological data at all.

*Anthropometry:* 18 studies detailed methods of anthropometric measurements. The World Health Organisation/ National Centre for Health Statistics were the most commonly used growth references.

*Other data:* Only one study measured psychological variables. Measurement of nutrition, cognitive development, socio-economic status and psychological variables most frequently used questionnaire based data collection.

### Loss to follow up

Attrition in SSA birth cohorts, measured from induction to last follow up, has a median of 28% ranging from 0% to 72.9%. Attrition rates were as follows: 0 to 10% attrition in 4 studies, 11–20% in 1 study, 21–30% in 4 studies, 31–40% in 3 studies and more than 40% in 4 studies. Twelve studies did not clearly report loss to follow up.

Problems reported by studies leading to high rates of attrition included: high infant mortality rates, family relocation, refusal to participate, maternal death, wave attrition (not present for one follow up, but return for the next) and failure of researchers to retrace study individuals.

Efforts to promote successful follow up included: providing participants with incentives – eg, ‘Birth To Twenty’ provided participants with basic mobile phones to aid contact; the Asembo bay cohort study provided free health clinics for all participants and a gift pack containing baby care items was given to mothers (this reduced attrition from 18.9% in 1992 to 7.7% in 1994). The Asembo Bay study also provided free medications, immunizations, and transportation to and from the study clinic, which contributed to a rate of loss to follow-up of only 4 percent over the first 18 months of follow up.

Other factors contributing to successful follow up included modern communication systems, close and frequent contact with all study sites for standardisation, short periods between phases of data collection and detailed contact information stored in computerised systems. These measures, however, were not appropriate or feasible for the majority of the studies reviewed.

## DISCUSSION

This review examined birth cohort studies in SSA – their methodologies and the challenges they face – in light of the absence of biobank studies in this region and with a view to making recommendations about future plans for this area of research. The study clearly shows that, although a number of efforts do exist, they evolved in their local settings and they weren’t planned or organized in any systematic way. Also, they very rarely (if ever) collect and store DNA material and frozen plasma for further genetic and biochemical studies.

### Study limitations

Attempts to overcome publication bias were made by searching for unpublished literature via OpenSIGLE (System for Information on Grey Literature in Europe) an open access source of bibliographical references of reports and other grey literature produced in Europe until 2005. It is, however, possible that some sources of grey literature were missed. Only two papers were retrieved in French, and none in Portuguese, despite the large Francophone and Lusophone populations in Africa. Although no language restrictions were applied, it may suggest the design of the search criteria was not optimised to retrieving these studies.

Due to the longitudinal nature of cohort studies large numbers of papers are produced, over long periods of time, from multiple rounds of study follow up. It is, as a result, challenging to capture the most updated description of an individual study. Efforts were made to group papers by study, both by hand (matching authors, years and sample sizes) and citation mapping, in order to identify the most recent description of any particular study and to capture all the data across the spread of publications arising from a single cohort. It is, however, possible that the current status of a study was not identified. Study characteristics are often reported differently between publications as cohort studies continue to develop over time in their methods and study sub-populations. It is possible that the conclusions of this study about the lack of good biological data collection reflect unreported data or failure to capture data, rather than a genuine lack of samples. Writing to authors may have helped to indentify the most comprehensive and current descriptions of birth cohort studies.

### Study quality

Comparability across birth cohort reviews is complicated by the different definitions of birth cohort studies. The definition is generally recognised as the longitudinal follow up of a group of people born at, or around, the same time. The descriptive elements considered important in this review include the study size at enrolment, the age of study participants at enrolment, the duration of follow up and the frequency of these follow ups. This review identified a subgroup of twenty eight studies that met strict criteria (minimum enrolment of 500 or more study participants with at least 12 months of follow up and enrolment at less than 60 months of age) with a view to identifying studies suitable for biobank data collection for the purposes of studying important causes of child health and development.

This review demonstrated, first, that the majority of birth cohort studies researched a specific sub-population rather than representative population samples eg, assessing HIV related outcomes in offspring of HIV positive mothers. Second, the SSA birth cohorts are largely restricted to small sample sizes with short periods of active follow up, with sample sizes varying from 571 to 11 342. Only two birth cohort studies were identified with study sizes greater than 5000. The ‘Birth to Twenty’ study in South Africa began following an unselected population of 3275 mothers and their children in 1990 and is still ongoing today. It is the best example of a large scale, long term birth cohort in SSA. However, it is of relatively small scale when compared to similar birth cohort studies in developed countries such as Avon Longitudinal study of Parents and Children (ALSPAC) which studied 13 971 births at induction (2). Typical genome wide association study sizes comprise several thousand, or even tens of thousands, of individuals. Recommended sample sizes for gene-environment interaction studies are of the order of twenty thousand participants with a specific outcome of interest (16). Small sample sizes of the African studies identified in this review are unlikely to provide a secure basis upon which to study the genetic and environmental influences of health and development outcomes in pregnancy and early childhood.

Second, the large majority of studies followed the cohort for less than two years, an insufficient amount of time on which to draw conclusions relating to the long term influence of developmental factors on future child, adolescent and adult health.

Third, there is no single coordinated definition of a birth cohort and measurements vary greatly between different studies. It is, therefore, difficult to combine study results or conduct meta-analyses of data due to inherent differences in birth cohort study design and measurement methods. Efforts have been made to bring together data from the largest low and middle income country birth cohorts as part of the work of the Consortium of Health Orientated Research in Transitioning Societies (COHORTS), but this initiative does not include the creation of a large biological resource (17). The five largest prospective birth cohorts with sample sizes of 2000 or more newborns and at least 15 years of follow up were included in this initiative and the data sets of these studies pooled. Some of the challenges identified by the COHORTS group included differences in variable definitions and measurement techniques; different ages for which data are available; and different time periods captured by each study. These differences between the studies resulted in restriction of their analyses to only those variables which were collected consistently across the cohorts. These limitations also apply to the SSA studies identified in this review.

### Biological data

Human genomic studies have revolutionized our understanding of disease and rapid progress has been made in high income countries with completion of the human genome project, emergence of genome wide association studies and the prospect of whole genome sequencing and pharmacogenomics (18,19).

Africa, where all human populations originated, is the most genetically diverse region in the world. To date, the relative risks (or odds ratios) for complex diseases associated with genetic loci – studied mainly in high-income countries – have been small (1.5 or less) (20). People of African origin display shorter linkage disequilibrium (LD) blocks, allowing for more precise mapping of loci associated with disease risk and the potential to discover disease causing variants which may previously have been masked by large LD blocks in European populations (21,22). Genetic factors do not account for chronic disease susceptibility alone, rather they interact with environmental exposures to determine disease risk (23). Africa’s genetic diversity, combined with its environmental diversity, unique life exposures and natural selection pressures presents many exciting possibilities for genetic research.

This review serves to highlight the lack of systematically collected birth cohort data on genetic, environmental and lifestyle factors underlying child health and development problems of SSA. Only one of the birth cohorts identified took DNA samples to establish a DNA bank, and this contained only 2200 individuals. The majority of DNA samples taken by studies were one-off measurements for diagnostic assessment of HIV or malaria status. The 473 GWA articles contained in the National Human Genome Research Institute catalogue were assigned weight according to country of origin in a study by Rosenburg et al. (24). These comparative weightings showed that the contribution of sub-Saharan African countries to genome wide association studies, even when all SSA country inputs are combined (0.34), is insignificant compared to those of high income countries (eg, 205.5 in USA, 68.15 in UK and 37.02 in Germany).

Overall, 13 of the 28 studies collected no biological samples of any sort reflecting either a primary interest in alternative data collection, or a simple lack of resources, manpower and laboratory facilities to do so.

The same technologies which are being used in developed world biobanks have the potential to generate new knowledge about communicable diseases amongst mothers and children in Africa. However, a gaping divide exists in clinical and genomic research capacity between SSA and higher income countries (21). DNA based studies require stringent quality criteria for complex processing and storage of samples, access to laboratories which are equipped with state-of-the-art facilities and run by well trained staff (22). The complexity of undertaking these studies could, however, foster local capacity building and drive innovation for new research opportunities and development in SSA.

Genome-based studies in developing countries present important ethical considerations (25). Valid consent must be obtained in a way that ensures an informed and voluntary choice can be made by study participants, regardless of their level of education and literacy. Protecting the privacy of study participants is an essential consideration as GWA studies have the potential to reveal stigmatising information about an individual or population which may be used for harm (26). Due to the lack of large scale genotyping facilities in most sub-Saharan African countries, samples may require storage and export for processing in high income countries (27). It is essential that a balance is struck between protection of study participant’s privacy and the need for data sharing and release in research (28). Strict guidelines on sample handling and destruction are often required limiting the ability for secondary analysis and reuse of archived samples (29). Obtaining ethical approval for genomic research in developing countries is understandably a complex and challenging process. However, these challenges can be successfully met as the experience in malaria research has shown (25).

### Challenges of longitudinal studies in low-income countries

Longitudinal studies pose unique methodological challenges to researchers. In birth cohorts two types of sample loss are reported: initial non-enrolment and attrition on follow up. Both have the potential to cause systematic bias in collection and interpretation of results. In the developing world, failure to trace individuals is reported as the most common cause of attrition (30). Lack of infrastructure, administrative centres, national databases and aids such as widespread patient identifiers in SSA pose a challenge to data collectors. High rates of migration also pose a challenge to longitudinal studies especially as the more educated, urban section of a cohort may be more likely to migrate, potentially resulting in a sample no longer representative of the original population from which it was taken.

Efforts to overcome attrition have included providing participants with incentives to continue with the study, however, there is a risk of subsequently conditioning the cohort such that they are no longer representative of the normal population. Other studies have used national census information, army enlistment days and systematic searching of all homes in the study area to retrace study participants.

Despite the methodological challenges faced by longitudinal cohorts in the developing world they are achievable and studies such as the Pelotas birth cohort in Brazil are testament to this (31). The study which began in 1982, measuring over 4000 variables for 5914 study participants, is one of the largest and longest running birth cohorts in the developing world and is still ongoing today. Household sampling, army enlistment and the low emigration rate in Pelotas limited attrition and follow up in 2005 retraced 77% of the original cohort.

### Funding

Most birth cohort studies report difficulty in attracting funding for initiating studies and then supporting multiple rounds of follow up. There is a particular need for multiple sources of funding if birth cohort studies are to collect biological samples for biobank data. The UK Biobank, a cohort of 500 000 people with a baseline assessment and 8-year follow up is projected to cost US$ 104 million (€ 72.5 million) (32). The UK Biobank was funded by Wellcome Trust (the UK’s largest independent medical research charity), the Medical Research Council, the Department of Health, the Scottish Government, British Heart Foundation, the Northwest Regional Development Agency and others.

One study estimated that the cost of setting up a ten year study similar to the UK Biobank in SSA with additional exposure measurements, intervention trials and research capacity building – would cost anywhere between, US$ 23.7 million (€ 16.5 million) for a cohort of 150 000 people across three countries or US$ 2.56 billion (€ 1.8 billion) for a cohort of 400 000 people across four countries (33). The cost per person per year for the UK Biobank estimate to US$ 26 (€ 18) compared to US$ 14–644 (€ 9.8–449) for a large scale SSA study. Such investments are substantial. They will, however, have a long term effect by revolutionizing the infrastructure, training and future development of academic and clinical research in SSA, and may subsequently stimulate economic development.

## CONCLUSION

This review identified a larger number of relevant studies from 28 sites in Africa (34-91). Only one birth cohort study which systematically collected DNA samples and related health data was identified but this was of a small scale. Investment in research training, infrastructure and pilot studies alongside the creation of ethical frameworks, quality assessment and locating long term sources of funding are just a few of the initial challenges that need to be addressed to establish and then ensure the sustainability of such biobanks in SSA. Governments and not-for-profit agencies have made large investments towards funding biobanks in high-income countries. We suggest that it is now time they turned their resources towards investing in the research capacity of SSA, and in doing so, investing in the future of mothers and children upon whom a large burden of avoidable mortality is centred.

## References

[1] Batty GD, Alves JG, Correia J, Lawlor DA (2007). Examining life-course influences on chronic disease: the importance of birth cohort studies from low- and middle-income countries. An overview.. Braz J Med Biol Res.

[2] Golding J, Pembrey M, Jones R (2001). ALSPAC – the AVON Longitudinal Study of Parents and Children. I. Study methodology.. Paediatr Perinat Epidemiol.

[3] McCarron P, Davey Smith G. Physiological measurements in children and young people, and risk of coronary heart disease in adults. In: Gates A, ed.: A lifecourse approach to coronary heart disease prevention scientific and policy review. The Stationary Office, London, 2003, pp. 49–78.

[4] Silva PA (1990). The Dunedin Multidisciplinary Health and Development Study: a 15 year longitudinal study.. Paediatr Perinat Epidemiol.

[5] Kuh D, Ben Shlomo Y. A lifecourse approach to chronic disease epidemiology. Oxford Medical Publications, Oxford, 2004.

[6] Lucas A, Fewtrell MS, Cole TJ (1999). Fetal origins of adult disease – the hypothesis revisited.. BMJ.

[7] Dawber TR, Meadors GF, Moore FE (1951). Epidemiological approaches to heart disease: the Framingham study.. Am J Public Health Nations Health.

[8] Barker DJ. Mothers, babies and health in later life. Churchill Livingstone, Edinburgh, 1998.

[9] Lawlor DA, Davey Smith G, Clark H, Leon DA (2006). The associations of birthweight, gestational age and childhood BMI with type 2 diabetes: findings from the Aberdeen children of the 1950s cohort.. Diabetologia.

[10] Ollier W, Sprosen T, Peakman T (2005). UK Biobank: from concept to reality.. Pharmacogenomics.

[11] Wright AF, Carothers AD, Campbell H (2002). Gene-environment interactions – the BioBank UK study.. Pharmacogenomics J.

[12] Black RE, Cousens S, Johnson HL, Lawn JE, Rudan I, Bassani DG (2010). Global, regional, and national causes of child mortality in 2008: a systematic analysis.. Lancet.

[13] You D, Wardlaw T, Salama P, Jones G (2010). Levels and trends in under-5 mortality, 1990–2008.. Lancet.

[14] Enserink M (2009). Global health. Some neglected diseases are more neglected than others.. Science.

[15] Sirugo G, Hennig BJ, Adeyemo AA (2008). Genetic studies of African populations: an overview on disease susceptibility and response to vaccines and therapeutics.. Hum Genet.

[16] Burton PR, Hansell AL, Fortier I, Manolio TA, Khoury MJ, Little J (2009). Size matters: just how big is BIG?: Quantifying realistic sample size requirements for human genome epidemiology.. Int J Epidemiol.

[17] Richter LM Victora CG Hallal PC Adair LS Bhargava SK Fall CH the COHORTS Group COHORT Profile: The Consortium of Health-Orientated Research in Transitioning Societies Int J Epidemiol 2011Apr 21. [Epub ahead of print] 10.1093/ije/dyq251 21224276 PMC3378468

[18] Hindorff LA, Junkins HA, Hall PN, Mehta JP, Manolio TA. A catalog of published genome-wide association studies. Available at: www.genome.gov/gwastudies Accessed: 25 March 2011.

[19] Manolio TA, Collins FS (2009). The HapMap and genome-wide association studies in diagnosis and therapy.. Annu Rev Med.

[20] Manolio TA, Bailey-Wilson JE, Collins FS (2006). Genes, environment and the value of prospective cohort studies.. Nat Rev Genet.

[21] Kraft P, Hunter DJ (2009). Genetic risk prediction – are we there yet?. N Engl J Med.

[22] Dalal S, Holmes MD, Ramesar RS (2010). Advancing public health genomics in Africa through prospective cohort studies.. J Epidemiol Community Health.

[23] Adeyemo A, Rotimi C (2010). Genetic variants associated with complex human diseases show wide variation across multiple populations.. Public Health Genomics..

[24] Rosenberg NA, Huang L, Jewett EM, Szpiech ZA, Jankovic I, Boehnke M (2010). Genome-wide association studies in diverse populations.. Nat Rev Genet.

[25] de Vries J, Bull SJ, Doumbo O, Ibrahim M, Mercereau-Puijalon O, Kwiatkowski D (2011). Ethical issues in human genomics research in developing countries.. BMC Med Ethics.

[26] McGuire AL (2008). Caulfield T, Cho MK. Research ethics and the challenge of whole-genome sequencing.. Nat Rev Genet.

[27] Elliott P, Peakman TC (2008). The UK Biobank sample handling and storage protocol for the collection, processing and archiving of human blood and urine.. Int J Epidemiol.

[28] Church G, Heeney C, Hawkins N, de Vries J, Boddington P, Kaye J (2009). Public access to genome-wide data: Five views on balancing research with privacy and protection.. PLoS Genet.

[29] Muula AS, Mfutso-Bengo JM (2007). Responsibilities and obligations of using human research specimens transported across national boundaries.. J Med Ethics.

[30] Harpham T, Huttly S, Wilson I, Wet T (2003). Linking public issues with private troubles: panel studies in developing countries.. J Int Develop.

[31] Victora CG Hallal PC Araújo C Menezes A Wells JCK Barros FC Cohort profile: The 1993 Pelotas (Brazil) birth cohort study. Int J Epidemiol 2008 37 704 709[REMOVED HYPERLINK FIELD] 17846051 10.1093/ije/dym177

[32] UK Biobank UK. Limited report and financial statements for the year ended 30 September 2010. Available at: http://www.ukbiobank.ac.uk/docs/Annualreport.pdf Accessed: 20 March 2011.

[33] Holmes MD, Dalal S, Volmink J, Adebamowo CA, Njelekela M, Fawzi WW (2010). Non-communicable diseases in sub-Saharan Africa: The case for cohort studies.. PLoS Med.

[34] Adair LS, Martorell R, Stein AD, Hallal PC, Sachdev HS, Prabhakaran D (2009). Size at birth, weight gain in infancy and childhood, and adult blood pressure in 5 low- and middle-income-country cohorts: when does weight gain matter?. Am J Clin Nutr.

[35] Alemu T, Lindtjorn B (1996). Growth velocity among preschool Ethiopian children.. Acta Paediatr.

[36] Asefa M, Hewison J, Drewett R (1998). Traditional nutritional and surgical practices and their effects on the growth of infants in south-west Ethiopia.. Paediatr Perinat Epidemiol.

[37] Asefa M, Drewett R, Hewison J (1996). An Ethiopian birth cohort study.. Paediatr Perinat Epidemiol.

[38] Carme B Guillo du Bodan H Lallemant M Infant and child mortality and malaria in the Congo. The trend in the suburbs of Brazzaville between 1981 and 1988. Trop Med Parasitol 1992 43 177 180[REMOVED HYPERLINK FIELD] 1470838

[39] Byass P, Fantahun M, Mekonnen W, Emmelin A, Berhane Y (2008). From birth to adulthood in rural Ethiopia: the Butajira Birth Cohort of 1987.. Paediatr Perinat Epidemiol.

[40] Carme B Guillo du Bodan H Molez JF Trape JF Retrospective study on the mortality of children under 5 in a rural district of the region of Brazzaville (People’s Republic of Congo). I. Rate and causes of mortality. Bull Soc Pathol Exot Filiales. 1984 77 104 114[REMOVED HYPERLINK FIELD] 6722959

[41] Chilongozi D, Wang L, Brown L, Taha T, Valentine M, Emel L (2008). Morbidity and mortality among a cohort of human immunodeficiency virus type 1-infected and uninfected pregnant women and their infants from Malawi, Zambia, and Tanzania.. Pediatr Infect Dis J.

[42] Cohen NM Clayden AD King B Cross-sectional-type weight reference values for village children under five years in Lesotho. Growth 1976 40 107 121[REMOVED HYPERLINK FIELD] 1261873

[43] Elguero E, Simondon KB, Vaugelade J, Marra A, Simondon F (2005). Non-specific effects of vaccination on child survival? A prospective study in Senegal.. Trop Med Int Health.

[44] Ellison GT de Wet T Matshidze KP Cooper P The reliability and validity of self-reported reproductive history and obstetric morbidity amongst Birth to Ten mothers in Soweto. Curationis 2000 23 76 80[REMOVED HYPERLINK FIELD] 11949296 10.4102/curationis.v23i4.753

[45] (2008). Griffiths PL, Rousham EK, Norris SA, Pettifor JM, Cameron N. Socio-economic status and body composition outcomes in urban South African children.. Arch Dis Child.

[46] Fegan GW, Noor AM, Akhwale WS, Cousens S, Snow RW (2007). Effect of expanded insecticide-treated bednet coverage on child survival in rural Kenya: a longitudinal study.. Lancet.

[47] Greenberg AE, Nsa W, Ryder R (1991). Plasmodium Falciparum malaria and perinatally acquired human immunodeficiency virus type 1 infection in Kinshasa, Zaire. A prospective, longitudinal cohort study of 587 children.. N Engl J Med.

[48] Hauspie RC, Pagezy H (1989). Longitudinal study of growth of African babies: an analysis of seasonal variations in the average.. Acta Paediatr Scand Suppl.

[49] Jones LL, Griffiths PL, Adair LS, Norris SA, Richter LM, Cameron N (2008). A comparison of the socio-economic determinants of growth retardation in South African and Filipino infants.. Public Health Nutr.

[50] Kristensen I, Aaby P, Jensen H (2000). Routine vaccinations and child survival: follow up study in Guinea-Bissau, West Africa.. BMJ.

[51] Kristensen IA, Olsen J (2006). Determinants of acute respiratory infections in Soweto-a population-based birth cohort.. Afr Med J.

[52] ter Kuile FO Terlouw DJ Kariuki SK Phillips-Howard PA Mirel LB Hawley WA Impact of permethrin-treated bed nets on malaria, anemia, and growth in infants in an area of intense perennial malaria transmission in western Kenya. Am J Trop Med Hyg 2003 68 Suppl 68 77[REMOVED HYPERLINK FIELD] 12749488

[53] Lesaffre E, Asefa M, Verbeke G (1999). Assessing the goodness-of-fit of the Laird and Ware model – an example: the Jimma infant survival differential longitudinal study.. Stat Med.

[54] Lindtjorn B, Alemu T, Bjorvatn B (1992). Child health in arid areas of Ethiopia: longitudinal study of the morbidity in infectious diseases.. Scand J Infect Dis.

[55] MacKeown JM, Cleaton-Jones PE, Edwards AW (2000). Energy and macronutrient intake in relation to dental caries incidence in urban black South African preschool children in 1991 and 1995: the Birth-to-Ten study.. Public Health Nutr.

[56] MacKeown JM, Pedro TM, Norris SA (2007). Energy, macro- and micronutrient intake among a true longitudinal group of South African adolescents at two interceptions (2000 and 2003): the Birth-to-Twenty Study.. Public Health Nutr.

[57] MacKeown JM, Cleaton-Jones PE, Norris SA (2003). Nutrient intake among a longitudinal group of urban black South African children at four interceptions between 1995 and 2000 (Birth-to-Ten Study).. Nutr Res.

[58] Maleta K, Virtanen SM, Espo M, Kulmala T, Ashorn P (2003). Seasonality of growth and the relationship between weight and height gain in children under three years of age in rural Malawi.. Acta Paediatr.

[59] Malhotra I, Dent A, Mungai P, Wamachi A, Ouma JH, Narum DL (2009). Can prenatal malaria exposure produce an immune tolerant phenotype? A prospective birth cohort study in Kenya.. PLoS Med.

[60] Martorell R, Horta BL, Adair LS, Stein AD, Richter L, Fall CH (2010). Weight gain in the first two years of life is an important predictor of schooling outcomes in pooled analyses from five birth cohorts from low- and middle-income countries.. J Nutr.

[61] McDowell I, King FS (1982). Interpretation of arm circumference as an indicator of nutritional status.. Arch Dis Child.

[62] McElroy PD Lal AA Hawley WA Bloland PB Kuile FO Oloo AJ Analysis of repeated hemoglobin measures in full-term, normal birth weight Kenyan children between birth and four years of age. III. The Asemobo Bay cohort project. Am J Trop Med Hyg 1999 61 932 940[REMOVED HYPERLINK FIELD] 10674673 10.4269/ajtmh.1999.61.932

[63] McElroy PD ter Kuile FO Hightower AW Hawley WA Phillips-Howard PA Oloo AJ All-cause mortality among young children in western Kenya. VI: the Asembo Bay cohort project. Am J Trop Med Hyg. 2001; 64 1-2 Suppl:18-27 11425174 10.4269/ajtmh.2001.64.18

[64] McElroy PD, ter Kuile FO, Lal AA, Bloland PB, Hawley WA, Oloo AJ (2000). Effect of Plasmodium falciparum parasitemia density on hemoglobin concentrations among full-term, normal birth weight children in western Kenya, IV. The Asembo Bay cohort project.. Am J Trop Med Hyg.

[65] Naicker N, Norris SA, Mathee A, von Schirnding YE, Richter L (2010). Prenatal and adolescent blood lead levels in South Africa: child, maternal and household risk factors in the Birth to Twenty cohort.. Environ Res.

[66] Nokes DJ, Okiro EA, Ngama M, White LJ, Ochola R, Scott PD (2004). Respiratory syncytial virus epidemiology in a birth cohort from Kilifi district, Kenya: infection during the first year of life.. J Infect Dis.

[67] Norris SA, Griffiths P, Pettifor JM, Dunger DB, Cameron N (2009). Implications of adopting the WHO 2006 Child Growth Standards: case study from urban South Africa, the Birth to Twenty cohort.. Ann Hum Biol.

[68] Ochola R, Sande C, Fegan G, Scott PD, Medley GF, Cane PA (2009). The level and duration of RSV-specifc maternal IgG in infants in Kilifi Kenya.. PLoS ONE.

[69] Oomen HA Jansen AA t’Mannetje W. Machakos Project Studies: Agents affecting health of mother and child in a rural area of Kenya. XIV. Growth pattern or rural Akamba pre-school children. Trop Geogr Med 1979 31 412 439[REMOVED HYPERLINK FIELD] 524452

[70] Pedro TM, MacKeown JM, Norris SA (2008). Variety and total number of food items recorded by a true longitudinal group of urban black South African children at five interceptions between 1995 and 2003: the Birth-to-Twenty (Bt20) Study.. Public Health Nutr.

[71] Rayco-Solon P, Moore SE, Fulford AJ, Prentice AM (2004). Fifty-year mortality trends in three rural African villages.. Trop Med Int Health.

[72] Reid G, Kielkowski D, Steyn SD, Botha K (1990). Mortality of an asbestos-exposed birth cohort. A pilot study.. S Afr Med J.

[73] Richter L, Norris S, Pettifor J, Yach D, Cameron N. (2007). Cohort profile: Mandela’s children: The 1990 birth to twenty study in South Africa.. Int J Epidemiol.

[74] Richter LM Norris SA Ginsburg C The silent truth of teenage pregnancies – Birth to Twenty cohort’s next generation. S Afr Med J 2006 96 122 124[REMOVED HYPERLINK FIELD] 16541552 PMC1913473

[75] Richter LM, Yach D, Cameron N, Griesel RD, De Wet T (1995). Enrolment into birth to ten (BTT): Population and sample characteristics.. Paediatr Perinat Epidemiol.

[76] Ryder RW, Kamenga M, Nkusu M, Batter V, Heyward WL (1994). AIDS orphans in Kinshasa, Zaire: Incidence and socioeconomic consequences.. AIDS.

[77] Ryder RW, Nsuami M, Nsa W, Kamenga M, Badi N, Utshudi M (1994). Mortality in HIV-1-seropositive women, their spouses and their newly born children during 36 months of follow-up in Kinshasa, Zaire.. AIDS.

[78] Sabet F, Richter LM, Ramchandani PG, Stein A, Quigley MA, Norris SA (2009). Low birthweight and subsequent emotional and behavioural outcomes in 12-year-old children in Soweto, South Africa: findings from Birth to Twenty.. Int J Epidemiol.

[79] Sheppard ZA, Norris SA, Pettifor JM, Cameron N, Griffiths PL (2009). Approaches for assessing the role of household socioeconomic status on child anthropometric measures in urban South Africa.. Am J Hum Biol.

[80] Simondon KB, Simondon F, Simon I, Diallo A, Benefice E, Traissac P (1998). Preschool stunting, age at menarche and adolescent height: a longitudinal study in rural Senegal.. Eur J Clin Nutr.

[81] Steyn K, de Wet T, Richter L, Cameron N, Levitt NS, Morrell C (2000). Cardiovascular disease risk factors in 5-yearold urban South African children-the Birth to Ten Study.. Afr Med J.

[82] Steyn K, de Wet T, Saloojee Y, Nel H, Yach D (2006). The influence of maternal cigarette smoking, snuff use and passive smoking on pregnancy outcomes: the Birth To Ten Study.. Paediatr Perinat Epidemiol.

[83] Thandrayen K, Norris SA, Pettifor JM (2009). Fracture rates in urban South African children of different ethnic origins: the Birth to Twenty cohort.. Osteoporos Int.

[84] van Eijk AM Ayisi JG Ter Kuile FO Misore AO Otieno JA Kolczak MS Malaria and human immunodeficiency virus infection as risk factors for anemia in infants in Kisumu, western Kenya. Am J Trop Med Hyg 2002 67 44 53[REMOVED HYPERLINK FIELD] 12363063 10.4269/ajtmh.2002.67.44

[85] van Heerden AC, Norris SA, Richter LM (2010). Using mobile phones for adolescent research in low and middle income countries: preliminary findings from the birth to twenty cohort, South Africa.. J Adolesc Health.

[86] Growth decelerations among under-5-year-old children in Kasongo (Zaire). I. Occurrence of decelerations and impact of measles on growth. Bull World Health Organ 1986 64 695 701[REMOVED HYPERLINK FIELD] 3492301 PMC2490970

[87] Vella V, Tomkins A, Borghesi A, Migliori GB, Ndiku J, Adriko BC (1993). Anthropometry and childhood mortality in northwest and southwest Uganda.. Am J Public Health.

[88] Vella V, Tomkins A, Borghesi A, Migliori GB, Oryem VY (1994). Determinants of stunting and recovery from stunting in northwest Uganda.. Int J Epidemiol.

[89] Vidulich L, Norris SA, Cameron N, Pettifor JM (2006). Differences in bone size and bone mass between black and white 10-year-old South African children.. Osteoporos Int.

[90] Whati LH, Senekal M, Steyn NP, Nel JH, Lombard C, Norris S (2005). Development of a reliable and valid nutritional knowledge questionnaire for urban South African adolescents.. Nutrition.

[91] Yach D, Cameron N, Padayachee N, Wagstaff L, Richter L, Fonn S (1991). Birth to ten: child health in South Africa in the 1990s. Rationale and methods of a birth cohort study.. Paediatr Perinat Epidemiol.

